# CAR-T Regulatory (CAR-Treg) Cells: Engineering and Applications

**DOI:** 10.3390/biomedicines10020287

**Published:** 2022-01-26

**Authors:** Motahareh Arjomandnejad, Acadia L. Kopec, Allison M. Keeler

**Affiliations:** 1 Horae Gene Therapy Center, University of Massachusetts Chan Medical School, Worcester, MA 01655, USA; motahareh.arjomandnejad@umassmed.edu (M.A.); acadia.kopec@umassmed.edu (A.L.K.); 2 Department of Pediatrics, University of Massachusetts Chan Medical School, Worcester, MA 01655, USA; 3 NeuroNexus Institute, University of Massachusetts Chan Medical School, Worcester, MA 01655, USA

**Keywords:** CAR Treg, autoimmune disease, transplantation, gene therapy, engineered Tregs

## Abstract

Regulatory T cells are critical for maintaining immune tolerance. Recent studies have confirmed their therapeutic suppressive potential to modulate immune responses in organ transplant and autoimmune diseases. However, the unknown and nonspecific antigen recognition of polyclonal Tregs has impaired their therapeutic potency in initial clinical findings. To address this limitation, antigen specificity can be conferred to Tregs by engineering the expression of transgenic T-cell receptor (TCR) or chimeric antigen receptor (CAR). In contrast to TCR Tregs, CAR Tregs are major histocompatibility complex (MHC) independent and less dependent on interleukin-2 (IL-2). Furthermore, CAR Tregs maintain Treg phenotype and function, home to the target tissue and show enhanced suppressive efficacy compared to polyclonal Tregs. Additional development of engineered CAR Tregs is needed to increase Tregs’ suppressive function and stability, prevent CAR Treg exhaustion, and assess their safety profile. Further understanding of Tregs therapeutic potential will be necessary before moving to broader clinical applications. Here, we summarize recent studies utilizing CAR Tregs in modulating immune responses in autoimmune diseases, transplantation, and gene therapy and future clinical applications.

## 1. Introduction

Regulatory T cells (Tregs) are a T-cell subset known for their immunomodulatory function. Expression of CD4, CD25, and the master transcription factor, forkhead box P3 (FOXP3), are the main characteristic markers of conventional Tregs. However, other regulatory immune cells with different properties such as CD8^+^ Tregs [1], or type 1 regulatory T cells (Tr1) [2] have been described. Tregs are divided into “natural” Tregs that develop in the thymus or “induced” Tregs that are generated in the periphery [3]. Regulatory T cells suppress immune responses through multiple mechanisms including direct interaction with other immune cells or by producing immunosuppressive cytokines such as interleukin-10 (IL-10) and Transforming growth factor beta (TGF-β) [4,5]. Tregs are powerful suppressive cells due to the intrinsic properties of broadly suppressing T cells with differing antigen specificity through bystander suppression and induction of other suppressive cells by infectious tolerance. In bystander suppression, antigen-activated Tregs can suppress colocalized conventional T cells regardless of their antigen specificity [6,7,8]. Moreover, through infectious tolerance, Tregs can convert conventional T cells into induced Tregs by secretion of immune suppressive cytokines TGF-β, IL-10 or IL-35 or by interacting with dendritic cells [9]. Hence, Tregs have been used to modulate immune responses in transplantation, autoimmune diseases, and gene therapy [10,11,12,13]. Adoptive transfer of polyclonal Tregs that contain multiple T-cell receptor (TCR) specificities and regulate T cells through antigen-independent bystander suppression has been performed in early clinical trials [14,15]. However, utilizing the polyclonal Tregs may result in nonspecific tolerance which is known to limit robust immune responses when necessary, such as in response to dangerous pathogens, and may also increase the patient’s risk of cancer [16,17]. Additionally, a major hurdle for nonengineered Tregs is their conversion into proinflammatory T helper 17 cells (Th17) cells in response to certain immunological environments [18]. Therefore, directing Tregs towards a desired antigen may boost the overall response and lower the risk of broad and systemic immunosuppression or generation of an inflammatory response.

Alternatively, preclinical studies have shown antigen-specific Tregs may be more efficient due to their homing ability towards the cognate antigen [19,20]. Antigen specificity can be conferred to regulatory T cells by transducing them with recombinant TCR [21,22,23]. TCR-engineered T cells recognize peptides from both intracellular and surface derived proteins. Moreover, TCR-T cells have high affinity to cognate major histocompatibility complex (MHC)-peptide and induce a potent immune synapse formation [24,25,26]. However, TCR-engineered Tregs are MHC dependent, and mismatch hybridization of the exogenous and endogenous chains limit their application [27]. Conversely, chimeric antigen receptor (CAR) technology offers a non-MHC-dependent approach with clinical efficacy achieved in B-cell malignancies [28,29]. Moreover, CAR Tregs are less dependent on IL-2 than TCR-Tregs [30]. CAR T cells utilize an extracellular antigen recognition domain from a single-chain variable fragment (scFv) of an antibody combined with an intracellular signaling domain. This combination allows for the construct to activate a T-cell response without interacting with the antigen in the context of MHC [31]. 

### 1.1. Engineering Antigen-Specific Tregs

Engineering cells to express CAR constructs is commonly accomplished through viral vector systems such as lentivirus [30,32], Gamma-retroviral [29], and adeno-associated viral (AAV) vectors [33]. Additionally, viral-free systems such as the Sleeping Beauty (SB) [34], or piggyBac transposon have been used to integrate CAR encoding DNA with favorable integration into the target genome [35]. Furthermore, Clustered regularly interspaced short palindromic repeats (CRISPR)- CRISPR associated protein 9 (Cas9) gene-editing technology, which allows for the insertion of DNA at specific locations directed by RNA, has also been used to engineer CAR T cells. CRISPR-engineered CAR T cells, which express CAR from an endogenous TCR locus remain active for longer periods than their virus-transduced counterparts [36].

**Figure 1:**
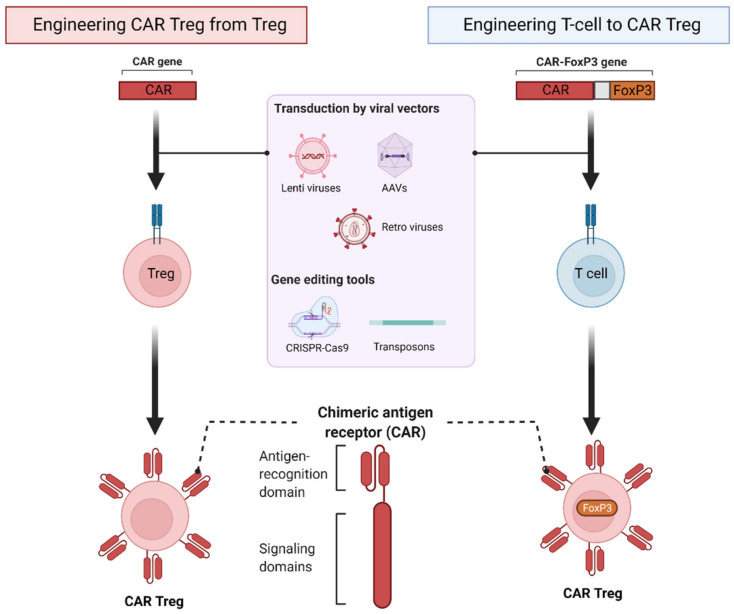
Generation of chimeric antigen receptor regulatory T cells (CAR Tregs). CAR Tregs are generated by transduction of polyclonal Tregs with CAR construct (left) or cotransduction of T cells with CAR construct and forkhead box P3 (FoxP3) gene (right).

The origin of transduced cells for the creation of CAR Tregs may be isolated polyclonal T cells, CD4^+^ T cells or Tregs (Figure 1). In CAR Tregs derived from polyclonal Tregs, Tregs are isolated and transduced with a CAR construct. However, this strategy is limited not only by low levels of Tregs in peripheral blood, but also the potential for downregulation of the Treg phenotype. Additionally, Tregs have been engineered by cotransducing CD4^+^ or CD3^+^ T cells with CAR constructs and FoxP3 cDNA. Studies have shown that transfection of T cells with FoxP3 induces regulatory activity [37,38]. This strategy aims to resolve the low population of primary Tregs in peripheral blood [39] and loss of FoxP3 expression in endogenous Tregs [18,37,40].

### 1.2. CAR-T Generations

CAR constructs are stratified into multiple generations by the combination of signaling domains, as shown in Table 1. The first-generation signaling domain only contains CD3ζ, whereas second and third generations include one or multiple additional costimulatory domains such as CD28 and/or 4-1BB, respectively [41,42,43,44]. Dawson et al. compared second-generation CAR constructs with multiple costimulatory domains. Their result suggested Tregs expressing CAR encoding wild-type CD28 are significantly more effective than other costimulatory domains in suppression of an immune response in a Graft versus hosed disease (GvHD) disease model [43]. Furthermore, Lamarthee et al. showed 4-1BB CAR Tregs exhibit decreased lineage stability and reduced in vivo suppressive capacities. Yet, transient exposure to mTOR inhibitors and vitamin C improves 4-1BB CAR Treg in vivo function [45]. In a separate study, Shrestha et al. showed CAR Tregs generated with the 4-1BB domain are able to prevent GvHD in murine model [46]. In addition, CAR T-cell studies also suggest 4-1BB costimulation will increase CAR T-cell persistence and ameliorate T-cell exhaustion [47,48]. Therefore, recent approaches created the third generation of CAR Tregs by including both 4-1BB and CD28 costimulatory domains [49,50]. Additional comparative studies are required to select the best combination for optimal and stable Treg functionality. Since the costimulatory domains and cell type used to generate CAR Tregs greatly determine their function, these attributes for each study described can be found in Table 2.

## 2. Application for CAR Treg Therapy 

Herein, we review the Treg application in modulating immune responses in multiple disease conditions focusing on the recent advancements of CAR Tregs.

### 2.1. GvHD

Graft-versus-host disease (GvHD) is caused by immune cells in the grafted tissues attacking recipient cells following allogeneic transplantation of organs rich in lymphoid cells, such as liver or nonirradiated blood transfusion [66]. Immune responses to solid tissue transplantation can be classified to direct and indirect pathways of allosensitization. In direct allorecognition, donor APCs migrate into the recipient lymphoid tissue where they present donor antigens to naïve T cells [67,68,69,70]. In indirect allorecognition, damage associated molecular patterns (DAMPs), which are released due to transplantation or cell death activate recipient APCs that migrate to draining lymph nodes (dLNs) where they engage recipient naive T cells, leading to T-cell activation [71,72,73].

In contrast to transplant rejection, which is mediated by the host immune response, graft-versus-host disease (GvHD) is caused by donor immunocompetent T cells primed by either donor or host APCs inducing an immune response against the host. The pathophysiology of GvHD can be acute or chronic depending on timing and range of symptoms [74].

Modulation of Tregs for therapeutic use has become an important area of investigation in GvHD, with the majority of studies focusing on in vitro-expanded polyclonal Tregs [75,76,77,78]. The first clinical trial of Tregs in GvHD showed modest impacts on acute GvHD symptoms but significant alleviation of the symptoms in chronic GvHD and reduction in the use of immunosuppressive agents [10]. Following trials demonstrated that adoptive transfer of ex vivo-expanded CD4^+^CD25^+^CD127^−^ Tregs prevented both acute and chronic GvHD [11,79,80]. Additionally, a pilot study of 10 liver transplant patients treated with ex vivo-generated Tregs showed normal graft function with few adverse events after withdrawal of immunosuppressive agents [81].

Subsequent studies began using CAR Tregs which were alloantigen-specific human Tregs engineered to express an HLA–A2–specific CAR (A2-CAR) [30]. These A2-CAR Tregs maintained high expression of FoxP3, CD25, Helios, and CTLA-4 in vitro and in vivo. Further, they prevented xenogeneic GvHD in immune-deficient NOD.SCID.γc −/− (NSG) mice, which was not observed in animals treated with polyclonal Tregs. Additional studies examined A2-CAR Tregs in skin allograft models that completely prevented rejection of allogeneic target cells and tissues in immune-reconstituted humanized mice in the absence of any immunosuppression [51]. Concurrently, Boardman et al. developed a second generation of CAR Tregs targeting the same antigen, HLA–A2. This A2-CAR showed antigen-specific suppression without eliciting cytotoxicity in vitro. Moreover, these A2-CAR Tregs migrated to HLA–A2-expressing cells and alleviated the alloimmune-mediated skin injury in mice [52]. Following the success of A2-targeting CAR Tregs, recent studies have focused on generating CAR Tregs with distinct targeting domains such as CD83 [46], which prevents GvHD in murine models, or CD19, which suppresses B-cell antibody production and pathology leading to GvHD [53]. In addition to the development of CD4^+^ CAR Tregs, CD8^+^ Tregs are emerging as potential candidates for suppression of GvHD [54]. 

These promising results have led to first CAR Treg clinical trial authorization by UK MHRA and US (NCT04817774) for kidney transplant patients, sponsored by Sangamo Therapeutics. This trial (STeadfast) utilizes CD4^+^/CD45RA^+^/CD25^+^/CD127^low/−^ Tregs that have been ex vivo engineered with a CAR construct to recognize HLA–A2 [82]. The STeadfast trial may support advantages of CAR Tregs over polyclonal expanded Tregs in a clinical trial setting, further expanding the possibility of using CAR Tregs in other disease conditions. Other biopharmaceutical companies are following close behind in hopes of starting clinical trials, such as Quell Therapeutics, focused on CAR Tregs for liver transplant recipients.

### 2.2. Diabetes

Type 1 diabetes (T1D) is an autoimmune disorder characterized by insulin deficiency due to the destruction of pancreatic β cells [83]. Several studies have focused on T1D due to its high prevalence rate (more than 1.6 million in the United States) [62]. Current therapies include insulin administration, diet and exercise [84]. Morbidity associated with T1D includes constant monitoring of blood glucose levels and lifelong insulin usage. 

Studies have shown reduced immune suppressive functionality of Tregs in patients with T1D [85,86]. This observation and the success of Treg transplantation in maintaining immunologic tolerance [87] has led to the application of Treg infusion in T1D patients to rescue remaining β cells. Infusion of expanded antigen-specific Tregs showed promising results in animal models in blocking and reversing diabetes [20,88,89]. Yet, isolating sufficient antigen-specific Tregs is challenging due to their rarity in circulation. Hence, studies have focused on transduction of the more abundant polyclonal CD4^+^ T cells with FoxP3 gene to convert them to Tregs. Although the ectopic expression of FoxP3 conferred a suppressor phenotype in naïve CD4^+^ T cells, this was not effective in diabetic mice. In contrast, FoxP3-transduced islet-specific T cells stabilized and reversed diabetes in vivo [90]. This suggested a marked benefit to antigen specificity, which alternatively could be conferred to polyclonal T cells by engineered TCR or CAR technology. In this context, Brusko’s group showed the ability of glutamic acid decarboxylase (GAD)-specific TCR-transduced Tregs to suppress the proliferation of both antigen-specific T cells and T cells with different antigen specificities in vitro [22]. Concurrently, Hull et al. transferred islet-specific TCRs to regulatory T cells and confirmed their ability to suppress the proliferation of CD4 and CD8 T cells in vitro [23]. Companies such as GentiBio and Abata are developing TCR-engineered Tregs for treatment of T1D. Since the application of TCR-transduced Tregs is limited by MHC restriction, other studies have focused on the development of CAR Tregs specific to other diabetes antigens. Tenspolde et al. created insulin-specific CAR Tregs by transducing CD4 T cells with second-generation CAR and FoxP3 gene. Insulin-specific CAR Tregs showed a similar phenotype to natural Tregs, and their function was confirmed in vitro by suppressing proliferation of effector T cells. Although insulin CAR Tregs did not prevent diabetes in NOD/Ltj mice, the cells were found in the spleen 17 weeks after infusion [40]. In another study, human pancreatic endocrine marker, HPi2-specific CAR Tregs, were generated by transducing Tregs with a second generation of CAR construct, but failed to maintain expansion due to tonic signaling [56]. In another study, CAR Tregs were designed against two immunodominant GAD65 beta-cell epitopes. Both CAR Tregs homed to pancreatic islets of humanized T1D mouse model 24 h after infusion. Moreover, the Treg population was significantly increased in the pancreas and spleen of the CAR Treg-treated groups compared to the control groups. CAR Treg-treated groups also showed lower blood glucose compared to the control groups [57].

Additionally, allogeneic islet transplantation is a promising cell-based therapy for T1D. However, host-mediated immune rejection is a limiting factor in broad application of islet transplantation [91]. In a recent study, poly lactic-co-glycolic acid (PLGA) microparticles (MPs) were engineered for the localized and controlled release of immunomodulatory TGF-β1. In vitro, the incubation of the particles with CD4^+^ T cells resulted in the induction of polyclonal and antigen specific Tregs. However, the presence of particles did not lead to significant graft protection in vivo [92]. Pierini et al. utilized the transduction of Tregs with a second-generation CAR construct to generate FITC-specific CAR Tregs (mAb CAR Treg). In this system, the CAR construct expresses an FITC binding domain allowing the use of any FITC-conjugated antibody to target the desired antigen. Utilizing mAb CAR in combination with FITC-conjugated antibodies targeting MHC class I proteins, they were able to prolong islet allograft survival in vivo [55]. These findings further indicate the ability of CAR Tregs to regulate the immune responses leading to T1D.

### 2.3. Rheumatoid Arthritis

Rheumatoid arthritis (RA) is the most common inflammatory arthritis characterized by synovial inflammation, hyperplasia, autoantibody production, cartilage and bone destruction, and systemic features, including cardiovascular, pulmonary, psychological, and skeletal disorders [93,94]. Multiple immune cell subsets are involved in the development of RA. Among them, the interactions between T cells and macrophages play an essential role [95]. The current therapy for RA includes disease-modifying antirheumatic drugs (DMARDs, methotrexate), anti-TNF-α, anti-CTLA-4 or small-molecule targeted DMARDs. However, these therapies are lifelong and accompanied with side effects and incomplete clinical response [96,97]. Therefore, inducing self-tolerance prior to serious tissue damage would be advantageous. Studies have investigated the benefit of increasing Treg numbers or improving Treg functionality [98,99,100]. Wright et al. utilized Tregs specific for ovalbumin (OVA) to suppress OVA-induced arthritis by generating either TCR-transduced primary Tregs or TCR-FoxP3-transduced CD4^+^ T cells, to induce the Treg phenotype [21]. In vitro, TCR-FoxP3 CD4^+^ T cells, but not TCR-Tregs, proliferated in response to antigen. Furthermore, both engineered Tregs showed OVA-dependent suppression of proliferation of T cells specific for a different antigen through bystander suppression. In vivo, both TCR-Tregs and TCR-FoxP3-induced Tregs localized into the damaged tissue, with neither converting to proinflammatory Th17 cells. Moreover, engineered Tregs reduced the number of inflammatory Th17 cells and significantly decreased arthritic bone destruction [21]. Utilizing T cells differentiated into Tregs targeting type II collagen, Sun et al. showed that Tregs differentiated from CD4^+^ T cells isolated from RA mice after onset of disease reversed collagen-induced arthritis (CIA) progression in mice. Moreover, these antigen-specific Tregs suppressed inflammatory cytokines and were stable in vivo [101]. In contrast, Raffin et al. applied the CAR technology to generate antigen-specific Tregs directed against citrullinated vimentin (CV), which is present abundantly in the extracellular matrix of inflamed joints in RA patients [58]. Sonoma Biotherapeutics is currently developing at CAR Treg therapy for RA.

### 2.4. Multiple Sclerosis

Multiple Sclerosis (MS) is an autoimmune demyelinating and neurodegenerative disease caused by autoreactive T cells recognizing myelin epitope, resulting in irreversible disability in more than 1 million people in the United States [102]. The treatment of MS includes nonspecific immune-suppressive drugs or B-cell-depleting monoclonal antibodies. However, the current treatments may result in severe side effects and cause global immune suppression, hence more specific and local treatments are needed [103,104]. Tregs in patients with MS have been found to secrete more IFN-γ and less IL-10 compared with healthy controls [105,106]. Considering the impaired function of Tregs in MS patients [107,108], utilizing Treg cell therapy has been suggested. 

Preclinical studies using an experimental autoimmune encephalomyelitis (EAE) model, a flawed but adequate murine model of MS, confirmed the effectiveness of Tregs in suppressing antigen-specific autoreactive immune responses [109] through a mechanism that involves IL-10 [110]. Expectedly, adoptive transfer of antigen-specific Tregs derived from TCR transgenic mice was successful in controlling a murine model of MS [111]. To achieve a greater number of cells for adoptive transfer, Fransson et al. modified CD4^+^ T cells with CAR targeting myelin oligodendrocyte glycoprotein (MOG) and murine FoxP3 gene to create antigen-specific Tregs. The MOG-CAR Tregs suppressed effector T cells’ proliferation in vitro. Moreover, the engineered Tregs localized into various regions in the brain after intranasal cell delivery. The MOG-CAR Tregs reduced disease symptoms and decreased proinflammatory cytokine mRNAs in brain tissue in EAE mice [37]. Later, Kim et al. engineered Tregs with myelin-basic protein-specific TCR that was derived from MS patients. These engineered Tregs upregulated Treg markers and were activated in response to the antigen. In vitro, they suppressed the effector T cells for that were specific for the same antigen and T cells with different antigen specificity through bystander suppression. In vivo, the TCR-Tregs localized in the brain and spinal cord and significantly reduced disease score in the EAE MS mouse model [112]. The application of immunomodulatory engineered Tregs in preclinical studies highlights their potential role in reducing morbidity and mortality associated with MS, with several biopharmaceutical companies are pursuing this such as Abata Therapeutics and TeraImmune. 

### 2.5. Inflammatory Bowel Disease

Inflammatory Bowel Disease (IBD) describes conditions characterized by chronic inflammation of the gastrointestinal tract. Ulcerative colitis (UC) and Crohn’s disease (CD) are the most common forms of IBDs. In CD, all layers of entire gastrointestinal tract can be affected by inflammation, whereas in UC inflammation occurs in colonic mucosa [113,114,115]. Common symptoms in CD include fatigue and abdominal pain, while in UC, bloody stool and diarrhea are most common [116]. Depending on the severity of the disease, treatments include nonsteroid anti-inflammatory (anti-TNF) drugs, steroids, and antibiotics [117]. 

Studies suggest the imbalance between gut microbiota and the immune response play an important role in the IBD [118]. Moreover, evidence suggests that Tregs have a crucial role in maintaining tolerance and preventing autoimmune disease. However, intestinal inflammation is not associated with a reduction in Treg population. Yet, mice with deficient Treg activity are more susceptible to developing severe colitis [119,120]. Therefore, multiple studies have attempted to harness the Treg suppressive activity to maintain tolerance in UC. In one study, isolated CAR Tregs against a known antigen of colitis (2,4,6-trinitrophenol (TNP)) from transgenic mice suppressed effector T-cell proliferation in vitro. In vivo, after induction of colitis, increased survival rate was observed in the CAR Treg transgenic mice compared with wild-type animals. Moreover, transfer of TNP-CAR Tregs into a colitis mice model reduced the symptoms and increased survival rates. The TNP-CAR Tregs were also able to localize in the inflamed colonic mucosa. In addition, TNP-CAR Tregs bystander suppressed the oxazolone-induced colitis [60]. In a subsequent study, they transduced murine Tregs with TNP-CAR which maintained FoxP3 expression and proliferated in response to the antigen ex vivo. In vivo, transfer of TNP-CAR Treg resulted in antigen-specific and dose-dependent amelioration of colitis [62]. Following these studies, CAR Tregs were generated against a different antigen, carcinoembryonic antigen (CEA), which is overexpressed in both human colitis and colorectal cancer. CEA-CAR Tregs were found in the colons of the diseased mice and suppressed the severity of the colitis compared to control animals [61]. 

Studies suggest an important role of IL-23 receptor (IL-23R) in the pathogenesis of autoimmune diseases including CD [121,122]. In addition, increased expression of IL-23R was shown in patients with CD compared to healthy controls. Therefore, in a recent study, Tregs were transduced with a second generation of CAR containing CD28 costimulatory domain targeting IL-23R. CAR Tregs suppressed conventional T-cell proliferation in vitro, homed to the target organs and reduced peak of disease and intestinal inflammation in mice [62]. Cell-based therapies may be a promising and effective modality for treatment or attenuation of UC and CD. 

### 2.6. Asthma

Asthma is a chronic respiratory disease affecting 300 million people worldwide [123]. The symptoms associated with asthma include wheezing, shortness of breath, chest tightness, cough and fixed airflow obstruction in severe chronic patients [124,125]. The standard of care for asthma includes anti-inflammatory and bronchospasmolytic drugs. However, 10–20% of patients are resistant to these symptomatic treatments [126]. Asthmatic patients are shown to have impaired and reduced number of Tregs [127,128]. Therefore, new approaches have focused on preventing airway inflammation by transferring regulatory T cells in a preclinical model of asthma [129]. Adoptive transfer of Tregs resulted in increased expression level of IL-10 [130]. Further, one study utilized T regulatory cell epitope (Tregitopes) to induce highly suppressive allergen-specific Tregs. Tregitopes are linear sequences of amino acids contained within the framework of monoclonal antibodies and immunoglobulin G that activate natural regulatory T cells [131]. 

Treatment with Tregitopes inhibited allergen-induced airway hyperresponsiveness and lung inflammation [132]. To direct Tregs towards asthma associated antigens, Skuljec et al. applied CAR technology. They engineered second-generation Tregs against CEA, a glycoprotein present on the surface of adenoepithelia in the lung and gastrointestinal tract, were isolated from transgenic mice; the same antigen used in the UC study described above [61]. They showed the activation and homing of the CEA-CAR Tregs in the inflamed lung of asthmatic mice. Moreover, the CEA-CAR Tregs ameliorated the inflammation to a greater degree compared to the nonmodified Tregs [63]. 

### 2.7. Vitiligo

Vitiligo is a skin disease characterized by progressive skin depigmentation with 0.5–1% frequency around the world [133]. Studies have shown that depigmentation is associated with the infiltration of T cells and macrophages to the dermis [134,135]. Further studies indicated that isolated T cells from patients’ skin are cytotoxic against melanocytes [136]. In addition, impaired Treg activity and decreased population of Treg were reported in Vitiligo patients [137,138]. Treatments include phototherapy, topical corticosteroids, calcineurin inhibitors, and depigmentation with p-(benzyloxy)phenol, or systemic treatment with corticosteroids, ciclosporin and other immunosuppressive agents [139]. Adoptive transfer of Tregs and use of rapamycin resulted in remission of the disease in mice [140]. These findings led researchers to generate CAR Tregs against ganglioside D3 (GD3), a surface marker overexpressed in melanocytes. The CD4^+^ FoxP3^+^ Tregs were transduced with the GD3-targeting CAR construct. In vivo, animals treated with CAR Tregs showed greater levels of IL-10, regulated cytotoxicity against melanocytes and delayed depigmentation compared to the group that received untransduced Tregs [59]. 

### 2.8. Hemophilia

Deficiency in Factor VIII (FVIII) or Factor IX (FIX), known as hemophilia A or B, respectively, are x-linked inherited bleeding disorders caused by mutations in clotting factor genes [141,142]. The current clinical treatment for hemophilia includes protein replacement therapy, which requires frequent administration of the coagulation factors and fails to completely prevent bleeds and joint damage [143]. Moreover, introducing coagulation factors, especially in patients with severe hemophilia, may provoke an antidrug immune response. To promote tolerance to coagulation factors, immune tolerance induction (ITI) through daily exposure to high-dose FVIII, has been used [144]. However, ITI is costly and not successful in all patients [145,146]. Several clinical trials have focused on gene therapy as a long-term and single-dose treatment to avoid frequent administration [147]. Other researchers have developed additional strategies utilizing Tregs to induce tolerance. Initially, Miao et al. confirmed the ability of FVIII-specific CD4^+^ FoxP3^+^ T cells to suppress FVIII antibody production in vivo [148]. Another study showed transferring ex vivo expanded polyclonal Tregs suppressed antibody formation against FVIII protein therapy even after the transferred cells became undetectable [13]. However, the need for a large number of Tregs and the risk of general immune suppression led researchers to the development of antigen-specific Tregs. Smith et al. enhanced specific Tregs by primming them with FVIII, which resulted in greater suppressive function compared to expanded naïve Tregs. However, the number of the FVIII-specific Tregs is a very small percentage of the total Treg population [149] even with expansion, hence other approaches are needed. While some groups are focused on engineering Tregs with an antigen-specific TCR [150], as well as companies such as TeraImmune, others utilized CAR technology [144]. Yoon et al. generated second-generation, FVIII-specific CAR Tregs from isolated Tregs that suppressed both B-cell and T-cell responses to FVIII. Their data also suggest bystander suppression by modulating proliferation of FVIII-specific T-effector cells with specificity for different FVIII domains [64]. In addition, Herzog et al. isolated FVIII-specific CD4^+^ T cells and transduced them with FoxP3, creating Tregs which showed suppression of FVIII antibody production [151]. Further, Fu et al. converted CD4^+^ T cells to FVIII-CAR Tregs by transducing them with a third-generation CAR construct and FoxP3 gene. Using this approach, they benefited from a greater number of cells and overcame the plasticity and transient nature of the adoptively transferred Tregs. Their data confirmed that the ectopic expression of FoxP3 creates effective and functional Tregs with the ability to inhibit antibody production against FVIII in vivo [50]. In a recent study, Rana et al. compared the functionality of FVIII-specific CAR and TruC (TCR fusion construct) Tregs. To generate TruC Tregs, they fused FVIII scFv to murine CD3ε and observed in vivo suppression with limited persistence. A second-generation CAR was compared to the TruC Tregs, and they observed loss of suppressive activity in CAR Tregs. Single amino acid mutations in CD3 or CD28 increased the Tregs’ persistence and changed cytokine profile, respectively, but did not restore tolerance [65]. 

Antigen-specific CAR Tregs are proving to be successful treatments to modulate the inhibitor formation associated with coagulation factors.

### 2.9. CAR Tregs in Gene Therapy

Recombinant adeno-associated virus (rAAV) is one of the most successful gene delivery tools with currently 2 FDA- and 1 EMA-approved treatments for a broad range of diseases. However, the immune responses observed in clinical trials have limited the therapeutic application. The T-cell responses against AAV capsid were first observed in a clinical trial using intravenous delivery of AAV for hemophilia, and led to the loss of transgene expression, a result never predicted by preclinical studies. To maintain transgene expression, immunosuppression is now widely used in AAV clinical trials [152,153,154,155,156]. Steroids are commonly used to modulate immune responses, but they do not specifically target the capsid-specific T cells and may also result in Treg depletion [157,158]. Interestingly, clinical studies suggest that the induction of local Tregs in gene therapy studies, specifically noted in intramuscular delivered AAV, enhance long-term transgene expression [159,160,161]. Induction of capsid-specific Tregs could enhance transgene expression and clinical outcomes of AAV gene therapy. In a novel study, administration of encapsulated rapamycin (ImmTOR) was codelivered with AAV gene therapy and resulted in reduced humoral and T-cell responses to capsid, immune infiltration, and stable transgene expression. Moreover, inductions of Tregs were shown to be a vital component of the ImmTOR response, as Treg depletion greatly inhibited immunomodulatory effect [162,163,164].

Arjomandnejad et al. therefore designed a third-generation CAR Treg that was specific for AAV capsid. AAV-CAR Tregs in vitro display phenotypical Treg surface marker expression, and functional suppression of effector T-cell proliferation and cytotoxicity. In mouse models, AAV-CAR Tregs mediated continued transgene expression from an immunogenic capsid, despite antibody responses, produced immunosuppressive cytokines, and decreased tissue inflammation [49]. 

In addition to the capsid-specific immune responses, immune response against the delivered transgene is another limiting factor for broad applications of gene therapy. Hence, various studies have investigated different strategies to inhibit these responses, including broad immunosuppression [165], administration of monoclonal antibodies [166] and utilizing tissue-specific promoters [167]. One study utilized polyclonal Tregs to induce tolerance against coagulation factors 8 and 9 in hemophilia protein replacement therapy [13]. However, due to the low cellular input and the possibility of nonspecific immune suppression, transgene-specific CAR Tregs were generated [50,64,65,151]. AAV-CAR Tregs directed against the AAV capsid were also shown to bystander suppress immune responses to the immunogenic OVA transgene, similarly mediating continued transgene expression, producing immunosuppressive cytokines, and reducing tissue infiltration [49]. These data suggest the AAV-CAR Treg can suppress both AAV capsid immune responses and vector-expressed transgene immune responses. 

Gene editing has the potential to revolutionize the gene therapy field, and with the discovery of the clustered regularly interspaced short palindromic repeats (CRISPR)–Cas9 system, the interest in the ability to precisely edit specific genes of interest has been enhanced [168]. However, studies have detected Cas9-associated humoral and cellular immune responses [169,170,171] and the prevalence of anti-Cas9 antibodies and T cells within the human population [172,173]. Although much success of CRISPR editing has been described for ex vivo therapies, numerous in vivo editing strategies have suggested immune responses to Cas9 may inhibit therapeutic effects [171,174]. Ferdosi et al. modified Cas9 protein to eliminate immunodominant epitopes by utilizing targeted mutations while still preserving its function and specificity [175]. In addition, Moreno et al. showed immune-orthogonal orthologues of Cas9 circumvent the immune response and allow for multiple dosing [176]. 

Regulatory T cells, due to their immunomodulatory role, are superior candidates to inhibit the immune responses against Cas9. A study by Wagner et al. found Cas9-specific Tregs in addition to effector T cells in human samples. They further reported Cas9 Tregs suppress Cas9-effector T cells’ proliferation and function in vitro [173]. To avoid systemic suppression, engineering Tregs with a Cas9-specific TCR or CAR is one of the possible strategies to overcome Cas9-mediated immune responses [177,178]. 

## 3. CAR Treg Limitations

Antigen-specific T-regulatory cells are powerful tools for immunosuppression, with a wide variety of uses, from treatment of autoimmune diseases, to modulation of immune responses, to gene therapy, and the first clinical trial is currently approved for kidney transplants (NCT04817774). Additional investments are being made in the platform across several companies focusing on creating engineered Treg products for the treatment of autoimmune and inflammatory diseases [179]. However, further optimization is required to increase Tregs’ suppressive function and stability, prevent CAR Treg exhaustion, and assess their safety profile. 

An additional hurdle to the widespread use of cell-based products is the cost. Even without engineering, cell-based products are expensive and require specialized equipment. For example, in the year 2020, a single-dose administration of CAR T cells in hematological malignancy is estimated to cost around USD 454,611 [180]. Therefore, the optimization of CAR Treg production and efficacy is necessary for widespread therapeutical use, and further optimization such as moving towards universal CAR Tregs is needed. One approach which may reduce the production cost is generation of allogeneic CAR Tregs by removing TCR to avoid GvHD. This off-the-shelf product could potentially treat many patients from a single batch of engineered CAR-T cells. They could also be used in patients where autologous therapy is not possible. Moreover, repeated dosing is feasible in the event of relapse [181]. 

### Promise of CAR Treg and Perspective

Despite the discussed limitations, there are significant advantages to utilizing engineered antigen-specific T cells, especially CAR Tregs. Compared to polyclonal Tregs, CAR Tregs are MHC independent, home to the target tissue due to antigen expression, and show enhanced suppressive efficacy. Furthermore, intrinsic properties of Tregs have provided a benefit to CAR Tregs over CAR T cells, including their ability to suppress T cells with different antigen specificity through bystander suppression and to induce endogenous tolerogenic cells through infectious tolerance [22,49,60,64,112]. In particular, in the case of AAV-CAR Tregs, a single product can not only suppress immune responses against various AAV capsid variants, but also vector-delivered transgenes, without having to create a new CAR construct for every capsid or vector-delivered transgene, creating a versatile therapy [49]. 

Expanded Tregs have shown promise as therapies for several autoimmune and inflammatory diseases, and may be effective in similar types of diseases. Companies are currently investigating lupus, systemic sclerosis, and inclusion body myositis among others. One candidate is Myasthenia Gravis (MG), which is a chronic autoimmune disease leading to muscle weakness and fatigability [182]. Current MG therapies include immunosuppression, which results in severe adverse effects [183]. One study showed Treg impairment in MG patients [184]. Later, Aricha et al. administered ex vivo-generated Tregs in an MG rat model, which led to inhibition of disease progression [185]. These findings suggest that MG may be a candidate for CAR Treg therapy. The neurodegenerative disease amyotrophic lateral sclerosis (ALS) is a promising potential candidate. Neuroinflammation plays a role in ALS but is usually attributed to microglia. However, Beers et al. reported that regulatory T cells from ALS patients show less suppressive activity ex vivo and their dysfunctionality is related to disease progression and severity [186]. Moreover, ex vivo expansion of Tregs and reinfusion in an ALS mouse model resulted in significant reduction in disease progression [187]. Further, the reinfusion of expanded Tregs from ALS patients with IL-2 resulted in increased Treg suppressive functions and reduced disease progression in human subjects [188]. These findings make ALS an ideal candidate to evaluate the efficacy of antigen-specific CAR Tregs. AZTherapies is currently testing a CAR-Treg approach for treatment of several neurodegenerative diseases. Additionally, SCM LifeScience and TeraImmune are developing CAR Tregs for atopic dermatitis using a virus-free gene delivery system. In addition, several studies have reported the role of Tregs in multiple fibrotic diseases including cystic fibrosis, systemic sclerosis, cardiac and liver fibrosis [189,190,191,192]. Therefore, utilizing CAR Treg may prove beneficial to ameliorate fibrotic diseases as the leading cause of mortality. Engineered Tregs have additionally been suggested to control immune responses to gene therapies. This dual-cell and gene therapy approach could vastly broaden the breadth of disease types engineered Tregs could treat. In AAV, gene therapy studies suggest that AAV-CAR Tregs can modulate the immune response to the vector and delivered transgenes, which may eliminate the use of immunosuppressive drugs. Moreover, vector readministration may be required and CAR Tregs are a potential candidate to suppress preexisting immunity [193]. Similarly, adoptive transfer of CAR Tregs directed towards Cas may modulate the preexisting immunity towards CRISPR gene-editing tools, allowing for exciting new therapeutic tools for genetic diseases. However, the combined costs of these two expensive therapies currently limits their therapeutic application.

## 4. Conclusions

Although the field of engineered Tregs is early in development, many important studies described here have generated great excitement in the field. To date, studies have largely focused on the preclinical characterization, mechanism of action, and efficacy of CAR Tregs, but clinical development is on the horizon and significant investment is being made by pharmaceutical companies. Further optimizations are required for the widespread use of engineered Tregs, yet they remain a powerful and promising modality. These engineered T cells are starting to enter clinical trials and may provide significant therapeutic benefit to numerous diseases reaching beyond transplantation and autoimmune diseases. 

## Figures and Tables

**Table 1:** Chimeric antigen receptor (CAR)-T Generations.

CAR Generation	Stimulatory Domain	Costimulatory Domain(s)	Graphical Representation	Functional Observations
1st	CD3ζ	none	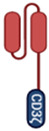	1st generation CARs are not used in CAR Treg studies as they are unable to activate resting T cells nor promote a continuous active response.
2nd	CD3ζ	CD28	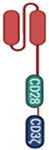	A CD28 costimulatory domain containing CAR showed the greatest function in GvHD mouse models when compared to 10 other signaling domains. These CARs can also show antitumor effects [43].
		4-1BB	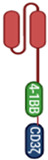	4-1BB-containing CARs are more resistant to T-cell exhaustion [48]. Function is improved by exposure to mTOR inhibitors and vitamin C [45].
3rd	CD3ζ	CD28 + 4-1BB	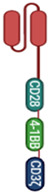	Designed to combine benefits of both CD28 and 4-1BB and increase functional capabilities of the CAR cells [49,50].

**Table 2:** Summary of applications of CAR Tregs in multiple disease conditions. *** indicates unknown.

*Disease Condition*	*Cell Type*	*CAR Generation*	*Target*	*Results*	*References*
*GvHD*	Human nTreg cells	Second generation (CD28)	HLA–A2 MHC complexes	Expression of regulatory cell markers and transcription factors in vitro and in vivo. Prevented GvHD in murine models.	Noyan et al., 2017 [51]
	Human CD4^+^CD25^+^ Treg cells	Second generation (CD28)	HLA–A2 MHC complexes	Antigen-specific suppression reducing alloimmune-mediated skin injury.	Boardman et al., 2017 [52]
	Human T cells	Second generation (41BB)	CD83+ dendritic cells	Prevented GvHD in murine models.	Shrestha et al., 2020 [46]
	Human Treg cells	Second generation (CD28)	CD19+ B cells	Suppressed GvHD associated antibody production.	Imura et al., 2020 [53]
	Human CD8^+^ CD45RC^low/−^ Treg cells	Second generation (CD28)	HLA–A2*02 MHC complexes	Suppressed immune responses caused by HLA mismatch. Human skin graft preserved in mouse models 100 days post engraftment.	Bézie et al., 2019 [54]
*Type 1 Diabetes*	Murine CD4^+^ FoxP3^+^ T cells	Second generation (CD28)	FITC mAB conjugate	Prolonged islet allograft survival.	Pierini et al., 2017 [55]
	Murine CD4^+^ T cells	Second generation (CD28)	Insulin	CAR Tregs remained in spleen 17 weeks post infusion.	Tenspolde et al., 2019 [40]
	Human CD4^+^ and CD8^+^ T cells	Second generation (CD28)	HiP2	Increased levels of IL-2 but limited expansion due to tonic signaling.	Radichev et al., 2020 [56]
	Murine Treg cells	***	GAD65 Beta cell epitopes	Localization to pancreatic islets 24 h post infusion. Large Treg population in the pancreas and spleen and lower blood glucose levels in CAR Treg treated groups.	Imam et al., 2019 [57]
*Rheumatoid Arthitis*	Human Treg cells	***	Citrullinated vimentin (CV)	Studies in progress.	Raffin et al. [58]
*Multiple Sclerosis*	Murine CD4^+^ T cells	Second generation (CD28)	Myelin oligodendrocyte glycoprotein (MOG)	Suppressed effector T-cell proliferation in vitro. In vivo, CAR Tregs localized to the brain and reduced levels proinflammatory cytokine mRNA and disease symptoms.	Fransson et al., 2012 [37]
*Vitiligo*	Murine CD4^+^ FoxP3^+^ Treg cells	Second Generation (CD28)	Ganglioside D3 (GD3)	Elevated IL-10, regulated melanocyte cytotoxicity, and delayed depigmentation.	Mukhatayev et al., 2020 [59]
*Inflammatory Bowel Disease*	Murine CD4^+^CD25^+^ Treg cells	Second Generation (CD28)	2,4,6-trinitrophenol (TNP)	Suppression of effector T-cell proliferation in vitro. Increased survival rate in vivo and reduced UC symptoms.	Elinav et al., 2008 [60] Elinav et al., 2009 [32]
	Murine CD4^+^CD25^+^ Treg cells	Second Generation (CD28)	Carcinoembryonic antigen (CEA)	Reduced severity of UC in murine models.	Blat et al., 2014 [61]
	Murine Treg cells	Second Generation (CD28)	IL-23R	Suppression of conventional T-cell proliferation in vitro. Reduced intestinal inflammation and reduced peak of disease.	121 ASGTC [62]
*Asthma*	Murine embryonic stem cells	Second Generation (CD28)	Carcinoembryonic antigen (CEA)	CAR Treg localization to the lungs and reduced inflammation.	Skuljec et al., 2017 [63]
*Hemophilia*	Human Treg Cells	Second Generation (CD28)	FVIII	Suppression of B-cell and T-cell responses and regulated FVIII-specific T effector cell proliferation.	Yoon et al., 2017 [64]
	Murine CD4^+^ T cells	Third Generation (CD28 + 41BB)	FVIII	Inhibited FVIII antibody production and maintained FVIII clotting ability.	Fu et al., 2020 [50]
	Murine CD4^+^CD25^+^ Treg cells	Second Generation (CD28)	FVIII	FVIII-specific CAR Tregs lost suppressive activity where TruC Tregs did not.	Rana et al., [65]
*Immune Response to Gene Therapies*	CD3^+^ T cells	Third generation (CD28 + 41BB)	AAV Capsid	Suppression of effector T-cell proliferation and cytotoxicity. Inhibition of capsid induced immune responses through increased immunosuppressive cytokines and reduced cellular infiltration. Transgene expression remained stable long-term in vivo. Isolated immune cell showed AAV capsid antigen specificity.	Arjomandnejad et al., 2021 [49]

## Data Availability

Not applicable.
